# Helium Plasma Effect on Breast Stromal-Enriched Lipograft: A Case Report

**DOI:** 10.1093/asjof/ojae087

**Published:** 2024-12-09

**Authors:** Aris Sterodimas

## Abstract

Autologous fat transfer for large volume augmentation, reconstructive, and cosmetic purposes has become more popular due to the inherent biocompatibility, accessibility, and low cost. For volume augmentation, the retention of grafted fat is unpredictable. Several approaches to autologous fat transfer have prepared the donor fat and/or the recipient-site increase fat graft retention as well as the predictability of the retention. This study is the first clinical report on the combination of radiofrequency helium plasma pretreatment of the recipient site and a cell-assisted lipotransfer technique for enhanced fat graft retention. One patient underwent autologous breast augmentation using the stromal-enriched lipograft technique to process the fat prior to injection. Only the right breast received pretreatment using radiofrequency helium plasma after infiltration. The processed fat was injected using a droplet style injector. The patient was followed for 2 years and underwent breast MRI examinations for measurement of the graft volume. The overall fat graft survival after 12 months for the stromal-enriched lipograft was 63%, whereas the survival for the stromal-enriched lipograft in combination with radiofrequency helium plasma was 89%. The addition of biostimulatory techniques to prepare the recipient site for breast augmentation enhanced the fat graft retention. Further clinical studies using radiofrequency helium plasma are required to justify using this modality as a recipient-site preparation technique.

**Level of Evidence: 5 (Therapeutic):**

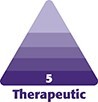

Over the last 30 years, there has been a constant interest in breast augmentation using autologous fat transplantation for reconstructive and cosmetic purposes. More recently, adipose tissue injection into the breast for lipoaugmentation had been associated with 2 limiting factors. First, fat injection in and around the breast could result in cyst formation, indurations, and fat necrosis.^1^ Second, the degree of reabsorption of the injected fat was unpredictable.^2^ To address these limitations, modern techniques aim to increase nutrient availability to the grafted adipose tissue by increasing angiogenesis and nutrient diffusion into the graft. These techniques consist of adipose tissue harvesting and washing, graft preparation through fat processing either mechanically or enzymatically, and finally fat injection.^3-5^ Additionally, the processed adipose tissue can be enriched with adipose-derived stem cells (ADSCs), platelet-rich plasma (PRP), and/or the stromal vascular fraction (SVF) to enhance fat graft retention by stimulating angiogenesis or the proliferation of progenitor cells.^6^ This technique is known as cell-assisted lipotransfer (CAL). More thorough reviews of current techniques to improve autologous fat graft retention have been published elsewhere.^7,8^ One example of CAL is the stromal-enriched lipograft (SEL), which was shown to increase fat graft retention by supplementing the lipoaspirate with the SVF from the processed adipose tissue.^9,10^ To further enhance the retention with CAL techniques, preparation of the recipient site should be considered. Biostimulatory techniques, such as low-level laser therapy, have been shown to increase blood flow and recruit immune cells after treatment.^11^ A recent study showed that preparation of the recipient site by treatment using a radiofrequency (rf) helium plasma device (Renuvion; Apyx Medical Corporation, Clearwater, FL) improved the viability of a bulk fat graft in a mouse model during the remodeling phase.^12^ Similarly, helium plasma was shown to increase the proliferation of ADSCs in vitro 72 h postexposure and stimulate ADSCs similar to PRP.^13-15^ Thus, this case report is the first to investigate rf helium plasma treatment of the recipient site and SEL for increased fat graft retention.

## CASE REPORT

**Figure 1.**
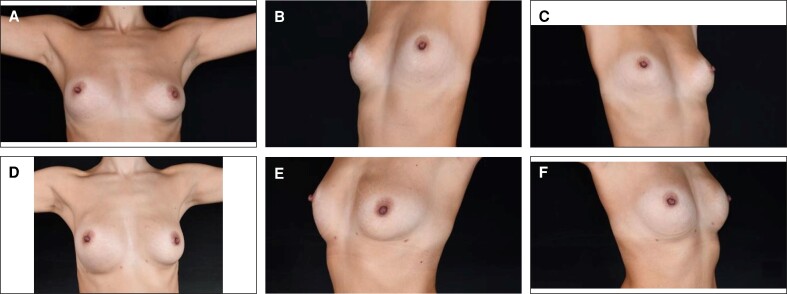
(A-C) Preoperative photographs of a 31-year-old female patient requesting breast augmentation with the use of autologous fat transplantation. (D-F) Postoperative photographs of a 31-year-old patient after 12 months of the SEL breast augmentation.

A 31-year-old female expressed the desire for a breast augmentation using the least invasive procedure to achieve an aesthetically accepted breast enhancement (Figure 1A-C). This study was IRB approved (Metropolitan General Hospital, IRB: 2022-12 A), and the patient signed the informed consent form for the proposed procedure. The patient was prescreened to exclude any family history with cancer, her preoperative BMI was 22.3, she breastfed during her only pregnancy, and she was a nonsmoker with no medical history including breast lumps or masses.

Prior to lipoaspiration, the flanks and abdomen were infiltrated with 500 cc of tumescent without anesthetic (500 cc normal saline with 1 mg of adrenaline). The fat tissue was harvested using a 4 mm cannula and the syringe method. The fat was processed following the SEL protocol.^16^ Briefly, the fat was processed enzymatically to separate the SVF from the bulk fat. Then, the SVF was centrifuged using the Automatic Cell Station (BSL, Seoul, Korea) at 1200*×g* 3× for 5 min each to separate the SVF and the enzyme. The pelleted SVF was then mixed with the purified fat and transferred to 20 cc syringes for fat injection. This fat-processing protocol was done inside the operating room in 55 min. Although not technically a closed system, the processed fat was never exposed to the air in the operating room. Prior to grafting the SEL, rf helium plasma was applied to the right breast only (Video). The incisions for treatment and fat grafting were located at the lateral part of the inframammary fold, at the midline of the inferior border of the areola, and at the midline of the superior border of the areola. The handpiece was deployed in the subcutaneous plane and activated only in a retrograde movement, that is, once engaged, the device was drawn backwards from the end of the treatment area toward the entry site of the breast. The emitted energy was fractional with 75% power, 1.5 L/min helium flow rate, and total 7.5 kJ energy applied. Two stab incisions were made to allow helium to escape the subcutaneous plane. Next, the SEL was injected subcutaneously using syringes with a 1.9 mm 4-hole cannula mounted onto the MAFT Gun (Dermato Plastica Beauty Co., Ltd, Kaohsiung, Taiwan), a droplet style injector allowing for precise volume control. As shown in Table 1, 210 cc SEL was injected in the right breast and 175 cc SEL in the left breast. The desired volume enhancement was determined with the patient preoperatively, and the total SEL added to each breast was estimated from the preoperative difference (30 cc). During the fat grafting, an additional 5 mL of SEL was added to the right breast for contouring.

**Table 1.** Preoperative and Postoperative Breast Volumes and fat Graft Survival Obtained by MRIs Before and After 12 Months of the Intervention

Breast	Volume pre-op (cc)	Volume post-op (cc)	Graft survival (%)
Right	190	377	89
Left	220	330	63

**Figure 2.**
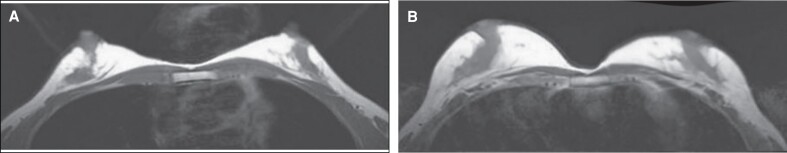
Preoperative (A) and 12 months postoperative (B) MRIs used for volume quantification.

The postoperative care included antibiotics (Ciprofloxaxcin, 500 mg every 12 h for 1 week), an analgesic (paracetamol 500 mg every 6 h for 3 days), and a breast garment for 1 month. The patient is shown 12 months after the procedure (Figure 1D-F). The patient underwent breast MRI examination (Magnetom Skyra 3T Tesla, MR VE11 software; Siemens, Malvern, PA) before and 12 months after the intervention, as shown in Figure 2. A blinded board-certified radiologist with advanced training in breast imaging interpreted both preoperative and postoperative results and quantified the volume using the transverse, posterior, and cephalocaudal dimensions (ImageChecker CAD software; Hologic, Bedford, MA). Soft tissue volume changes of the breast MRI were obtained, and the results are shown in Table 1. The overall fat graft survival after 12 months for the SEL and the SEL with rf helium plasma was 63% and 89%, respectively. The patient's postoperative BMI was 22.5. Patient satisfaction was assessed using the BREAST-Q (Memorial Sloan-Kettering Cancer Center, New York, NY, and University of British Columbia, Vancouver, Canada) preoperatively (Score 62) and postoperatively (Score 81). It revealed a high satisfaction with breast appearance and overall outcome.

## DISCUSSION

The benefits of autologous fat grafting for cosmetic and reconstructive applications allow physicians to provide a more satisfactory outcome for patients. This motivation drives further investigations into reducing the limitations of unpredictable retention and other adverse complications. The rationale behind CAL techniques like SEL is that aspirated fat is poor in progenitor cells, growth factors, and cytokines but can be enriched with SVF, PRP, and ADSCs.^9^ Although cultured ADSCs could be used to enrich fat grafts, they undergo rapid senescence in vitro and are difficult to culture while ensuring that they maintain their stemness.^17^ Successful fat graft retention requires a sufficient concentration of ADSCs to replace mature adipocytes due to their ability to resist the hypoxic and physical stress of fat grafting that inhibit mature adipocyte viability.^18^ Although it still needs to be investigated clinically, several mouse studies have shown that the majority of healthy adipocytes after remodeling originate from ADSCs in the donor fat and that revascularization occurs from recipient blood vessels growing into the graft.^19,20^ These ADSCs differentiate to replace the dead adipocytes and promote angiogenesis through growth factor secretion and neovascular differentiation. Fat graft survival is further improved in the presence of growth factors and cells in PRP and SVF.^21^

The addition of recipient site preparation is complimentary to SEL. Energy-based device treatment of the recipient tissue, such as low-level laser therapy, was shown to proliferate ADSCs through changes in their redox state and release of proangiogenic and proliferative factors.^11,22^ Helium plasma treatment of ADSCs promoted proliferation, cytokine and growth factor release, and induced epigenetic modifications similar to exposure to PRP in vitro.^13-15^ Although the donor fat is not treated in the presented case, the resident ADSCs and adipose tissue are treated and could potentially promote the release of cytokines and growth factors. The pretreatment of the recipient site using rf helium plasma treatment could stimulate an enhanced immune response from recipient site, potentially augmenting the number of immune cells and progenitor cells in the SEL through migration of recipient tissue cells.^23,24^ Initial SEL studies were documented to significantly increase the fat graft retention over unprocessed fat.^9,25^ Here, fat grafting was enhanced with the addition of rf helium plasma; however, the mechanisms need further investigation.

The main limitation of this study is that only 1 patient was treated; thus, any statements on safety, efficacy, or mechanism due to rf helium plasma need to be validated with a large-scale randomized controlled study. The comparison between fat retention outcomes using different volumes of SEL is a limitation for both fat retention metrics and definitively stating that the difference in retention is solely due to rf helium plasma. However, using equal volumes may have compromised patient satisfaction postoperatively. Additionally, more studies on the effects of rf helium plasma for this application are needed, because the supporting literature for plasma-induced biological effects is mostly observed in vitro or animal models. Furthermore, other energy-based devices, for example, fractional laser, should be investigated to compare the effects of rf helium plasma. Finally, another limitation is that the SEL method for processing the fat and treatment with the rf helium plasma is expensive and will need to be more widely accessible.
